# Evaluation of a Virtual Tai Chi Program for Older Veterans at Risk of Loneliness or Physical Deconditioning: A Quality Improvement Project

**DOI:** 10.3390/geriatrics9040091

**Published:** 2024-07-09

**Authors:** Bonnie D. Dawson, Hallie E. Keller, Linda M. Sawyer, Shannon Gorman, Jerome A. Sabangan, Adam McPartlin, Sarah Payne, Karl J. Brown, Gail Li, Dennis H. Sullivan

**Affiliations:** 1 Geriatric Research Education and Clinical Center, Central Arkansas Veterans Healthcare System, 2200 Fort Roots Drive, North Little Rock, AR 72114, USA; hallie.keller@va.gov (H.E.K.); linda.sawyer1@va.gov (L.M.S.); dennis.sullivan3@va.gov (D.H.S.); 2 VA Palo Alto Health Care System, 3801 Miranda Ave, Palo Alto, CA 94304, USA; shannon.gorman2@va.gov (S.G.); jerome.sabangan@va.gov (J.A.S.); 3 VA Puget Sound Health Care System, 1660 S. Columbian Way, Seattle, WA 98108, USA; adam.mcpartlin@va.gov (A.M.); sarah.payne@va.gov (S.P.); karl.brown2@va.gov (K.J.B.); ge.li@va.gov (G.L.); 4 Donald W. Reynolds Department of Geriatrics, University of Arkansas for Medical Sciences, 4301 W. Markham St., Little Rock, AR 72205, USA

**Keywords:** older adults, digital technology, telehealth, Tai Chi, virtual, VA Video Connect (VVC), physical activity, exercise

## Abstract

This Quality Improvement project evaluated the implementation of a virtual Tai Chi program for older Veterans (OVs) at risk of loneliness and/or physical deconditioning. A 12-week Tai Chi course was conducted virtually at three Veterans Affairs sites using VA Video Connect (VVC). Changes in physical function based on the 30-Second Chair Stand (30CST) and loneliness based on the De Jong Gierveld Loneliness Scale (DJGS) were measured, as were the OVs’ satisfaction and adherence. Of 109 OVs who enrolled, 74 completed the program with a mean attendance rate of 84%. Completers demonstrated a statistically significant improvement in the 30CST, and those who were moderately or severely lonely at baseline saw a statistically significant improvement in the DJGS. Course evaluations were generally very positive. Results suggest that a virtual Tai Chi program is an effective and very satisfying intervention for OVs at risk of loneliness or physical deconditioning.

## 1. Introduction

While COVID-19 was circulating earlier, the World Health Organization declared COVID-19 a pandemic on 11 March 2020 [1]. Faced with this global pandemic, the United States government as well as other public and private entities across the world issued guidelines and implemented restrictions to decrease exposure to and spread of COVID-19 [2,3,4]. Unfortunately, these restrictions also limited the physical and social interactions of many people, including older Veterans (OVs) [5,6,7,8,9]. This led to many older adults developing feelings of loneliness or becoming more sedentary and physically deconditioned during the pandemic [6,7,8,9,10,11,12,13,14,15]. As OVs have more comorbidities and less resilience compared to age-matched non-Veterans [16,17], they were particularly susceptible to developing such adverse effects [7,8,11,12,13,14,15,18]. Possibly due to their many health problems, sedentary lifestyle, and/or weak social support systems, many older Veterans remained at high risk for progressive deconditioning and loneliness even after all COVID-19 restrictions were removed [12,19,20]. This represented a serious public health concern. Being sedentary, which can rapidly lead to deconditioning [21,22,23], is associated with increased all-cause mortality, physical disability (i.e., an inability to independently perform all activities of daily living), worsening glucose control, osteoporosis, and greater risk of falls [19,24,25,26]. Loneliness can also be a devastating condition for older adults, including OVs. It is associated with a greater risk of cardiovascular disease, dementia, stroke, depression, anxiety, suicidality, and premature death [20,27]. For these reasons, interventions to prevent or ameliorate physical deconditioning and loneliness in this population were needed. Ideally, any such intervention needed to be relatively easy to implement, readily available, appealing to OVs, and effective at addressing the loneliness and deconditioning that OVs were experiencing during and after the COVID-19 pandemic. Tai Chi was known to be an intervention with such attributes. There was strong evidence indicating that the practice of Tai Chi was associated with many health benefits, including improvements in physical conditioning, better quality of life, and decreased loneliness [12,18,28,29,30,31,32,33,34]. Based on our experience and that of other centers, it was also known that many OVs enjoyed participating in Tai Chi classes and were eager to do so on a recurring basis [29,35,36,37]. Most of our experience in this realm, however, was in providing OVs the opportunity to participate in group classes where most of the participants attended in person. As it was important that OVs continue to observe social distancing recommendations during the COVID-19 pandemic, there was a need to provide the Tai Chi classes in a format that maximized safety while preserving the program’s effectiveness. Our previous experience and that of others [33,36,37,38,39], and the rapid increase in the use of video telehealth within the VA [2,3,4,40], indicated this could be accomplished by providing Tai Chi classes in a virtual format. There was also strong evidence that home-based exercises were effective at improving physical function [41]. For these reasons, a Quality Improvement (QI) project was initiated to provide a virtual Tai Chi program to OVs. 

The goals of the project were to determine if it was feasible to establish the program at three different VA healthcare facilities (stations) and to determine if participation in the program would yield physical benefits, decrease feelings of loneliness, and provide an enjoyable experience for OVs. Feasibility was assessed using several criteria, including the following: (a) after initial training, participants could successfully connect via VVC and actively participate in an entirely virtual group Tai Chi class; (b) a majority of the participants were able to subsequently complete at least five weeks of class by VVC with little to no technical difficulties; (c) no participants experienced safety concerns as a result of their virtual participation; and (d) survey results obtained from the participants were generally positive and did not indicate any strong objections or concerns about virtual classes. This report covers the time since the program was initiated in June 2021 until September 2022.

## 2. Methods

### 2.1. Participants

The virtual Tai Chi program was open to United States uniformed services Veterans aged 60 years or over who were enrolled at any of the three participating VA sites, including Little Rock, Palo Alto, and Puget Sound, and was conducted virtually using VA Video Connect (VVC), the VA’s secure videoconferencing platform [42]. To qualify for enrollment, the Veteran had to be capable of standing (with or without an assistive device), have the physical and mental capacity to perform basic Tai Chi exercises, and be comfortable actively participating using VVC. As this was a QI project, there was no a priori hypothesis-driven sample size calculation. An Institutional Review Board request for review determined that this activity did not meet the Common Rule or definition of research but did meet the criteria for operational improvement activities exempt from their review. Standards for Quality Improvement Reporting Excellence (SQUIRE) guidelines were followed in formulating this report [43]. 

### 2.2. Procedures

The program consisted of Tai Chi classes that met using VVC for one hour twice weekly for a total of 12 weeks. At all three sites, OVs were referred to the respective programs by their healthcare providers using an established consult system. At the Little Rock and Puget Sound facilities, OVs were also allowed to self-refer by calling one of the project coordinators. The schedule of enrollment differed by site. Some sites set fixed start dates for a class and all enrollees started and ended the 12-week course at the same time. Other sites used rolling enrollment where new participants entered the program at scheduled intervals. At sites using rolling enrollment, OVs were allowed to make up for missed classes by extending participation beyond the 12 weeks. Puget Sound used only fixed enrollment and Little Rock used rolling enrollment. Palo Alto used both enrollment methods, depending on the class instructor. 

When necessary, a digital divide consult was provided for any OV who wished to join the virtual Tai Chi program, did not have a device to support virtual participation, and met the digital divide eligibility criteria. Once approved, the digital divide consult provided Veterans with a government-issued tablet that was already set up to use VVC. Veterans were also provided assistance obtaining access to broadband services if it was needed. The tablet was on loan to the Veteran as long they continued to meet the eligibility criteria.

Before the enrolled Veterans attended their first virtual Tai Chi class, project staff queried them about their comfort level using VVC. For those OVs who were not facile using VVC, the project staff conducted practice VVC calls with them. These participants generally required about 30 to 45 min of training, provided in one or two virtual sessions, and possibly a brief telephone call to help them connect for the first time. All participants, once comfortable using VVC, received a brief VVC call to ensure their home environment and devices were set up appropriately so that they could see and hear and be seen and heard by the instructors. All class participants joined the classes using a desktop or laptop computer, tablet, or smart phone. When necessary, accessories were provided to allow the participant to connect their device to a larger screen (e.g., connect a smart phone to a television using a lightning port and HDMI cable).

### 2.3. Assessments

Once they were ready to use VVC, participants completed baseline assessments prior to or shortly after joining their first virtual Tai Chi class. This baseline assessment included demographic data, the EASY Health Screening tool to identify any contraindications or potential problems with participation [44], a 30-Second Chair Stand Test (30CST) [45], and the De Jong Gierveld Loneliness Scale (DJGS) [46]. The EASY Health Screening tool consisted of 6 questions, each of which asked about the presence of a different condition or symptom that could indicate an increased risk of adverse cardiovascular outcomes during exercise. Any positive response would either exclude the individual from the program or require an appropriate medical evaluation to clear the individual to join the program. The 30CST measures the number of times a patient can stand from seated, with arms crossed, within a span of 30 s. DJGS scores range from 0 to 11, with larger numbers indicating greater loneliness. These instruments were chosen for several reasons, including the fact that they had previously been validated for use in older populations and could be easily administered virtually. The 30CST is a very good indicator of physical performance as it is strongly correlated with multiple other markers of physical performance and physical function [47,48,49,50]. Prior studies have also demonstrated that both the 30CST and DJGS had adequate sensitivity to detect whether practicing Tai Chi induced a significant change in the measured parameter. Some participants completed additional assessments, but these are not the focus of this report. Participants were also given the VA group telehealth agreement stating the rules and expectations of VA staff and Veterans when attending group appointments virtually. The participants needed to concur with this agreement to continue in the program. When program staff scheduled each virtual class through VVC, participants received an email which contained the link to join the class. OVs were considered formally enrolled in the program after completing the baseline assessment and attending one virtual Tai Chi class.

Once a participant attended 24 classes, had to leave the program earlier than expected, or the course ended, the participant was asked to complete a final assessment, which included several of the same instruments used at initial enrollment. When completing the course assessments, OVs were also asked to complete a course evaluation survey. Attendance was recorded at each class session at all sites. Throughout the program, attendees were asked to tell us if they had sustained an injury or were experiencing any problems since joining the virtual Tai Chi course.

### 2.4. Intervention

Each of the three VA facilities that participated in the program scheduled virtual group classes independent of the other participating sites (i.e., there were three independent virtual programs). At all sites, each class was led by one or two staff members including one certified Tai Chi instructor. The instructor and participants from a given site all joined that site’s virtual group class via VVC. The participants joined the virtual class from remote locations, such as their home, and the instructor joined from the given VA facility. The additional project staff members, when available, were also on site and provided OVs with assistance in resolving technical difficulties or other urgent matters during each virtual class. The virtual Tai Chi classes were conducted at each center using the same standardized format. At all sites, a lesson plan that included welcome and announcements, a warm-up, drill of the day, and cool down, was followed by each instructor as described by Sawyer et al. and Brown et. al. [29,37]. Each instructor was trained to implement this lesson plan using a stepwise progressive teaching method. Participants were also provided other training materials (including links to online Tai Chi classes) and encouraged to practice between classes; however, this was not monitored.

Although VVC allows each participant to turn off their own microphone or camera, the video feed for every device was required to be turned on for the duration of the class. Project staff often managed microphones for all participants due to the physical distance (5–10 feet) between the participant and their device. When participants joined the group class, microphones were left open during the welcome and announcement time. Microphones were then muted to prevent any sound interruption during instruction and practice times. Participants used nonverbal communication such as a thumbs up or raising a hand when needing to have their microphone opened. Microphones were unmuted at the end of class to allow for additional questions, comments, and open discussion. Project staff also managed the view of the class. For most of the class, the instructor was spotlighted as the main image on the screen, with participants shown at the bottom of the screen in a smaller format. The spotlighted individual could be changed as needed, or two individuals could be spotlighted, which split the main screen in two. To ensure that the staff were able to maintain a clear view of all participants throughout each class, class size was limited to a maximum of 15 participants (including the staff).

### 2.5. Statistics

Descriptive statistics were used to summarize Veterans’ demographic characteristics, attendance, and rate of program completion, which was an indicator of feasibility. Veterans who attended at least 10 classes (equivalent to 5 weeks) and completed both the baseline and final assessments were considered program completers. The rationale for using 10 classes to define program completers was based on findings from prior research demonstrating that clinically meaningful changes occur within this timeframe. For example, Lee et al. found that substantial improvements (≥50%) in measures of physical function appeared after 5 weeks of Tai Chi participation among patients with knee osteoarthritis [51]. We also assumed that any changes in test scores observed among those with less than five weeks participation were probably unrelated to the Tai Chi program. Chi-square, Fisher’s exact, or *t*-tests were used to compare demographics of program completers with non-completers after excluding those with unknown demographic values. 

The change in DJGS score (final–baseline) was examined using a stepwise general linear model (GLM) controlling for baseline DJGS score, site, age, and percent of classes attended (<80% vs. ≥80%). In our GLM analysis, we treated the change in DJGS as a continuous measure. We transformed the baseline and final score to a three-level categorical variable (“not lonely”; “moderately lonely”; “severely lonely”) according to van Tilburg et al. [52]. To gauge further the potential clinical and statistical significance of the change in DJGS scores, the frequency with which OVs moved from one loneliness level to another between baseline and final testing was also examined using Krauth’s exact test of symmetry. 

The change in the 30CST (final–baseline) was examined using a stepwise GLM controlling for baseline 30CST, site, age, and percent of classes attended (<80% vs. ≥80%). Baseline and change in 30CST were both treated as continuous measures. 

The results of the course evaluation survey were summarized using descriptive statistics. Veterans rated their agreement with five statements on a Likert scale, with 1 representing lowest agreement and 5 representing highest. Ratings of the 5 statements were also averaged for a final score. Veterans also provided open-ended comments, which were summarized using qualitative thematic coding. 

Analyses were conducted using SAS^®^ Studio 3.71 and Excel v2308. Statistical significance was defined as two-tailed *p* < 0.05.

## 3. Results

### 3.1. Demographics and Program Adherence

**Figure 1:**
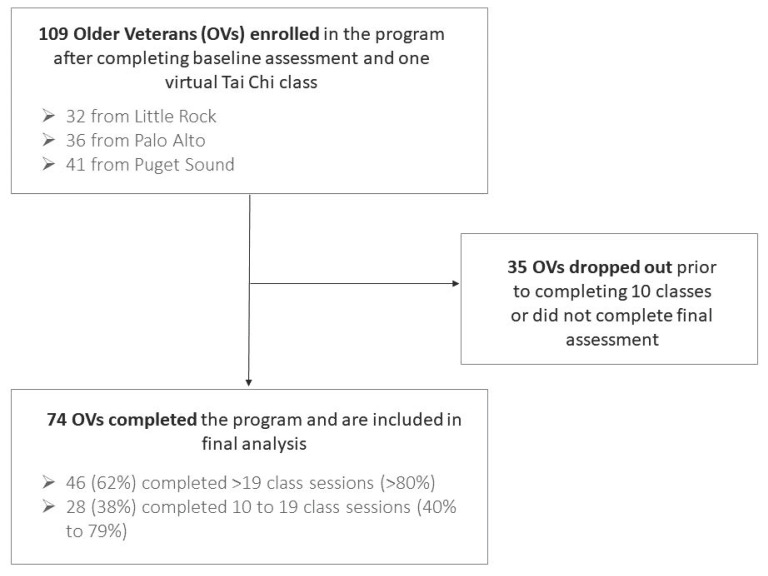
Summary of recruitment process.

One hundred and nine OVs formally enrolled in the program after completing the baseline assessment and attending one virtual Tai Chi class. No enrollee failed the EASY Health Screening. Based on our definition (see the Section 2), 74 (67.9%) of the 109 enrolled OVs completed the program after attending a mean 20.1 of 24 possible classes (Table 1). The remaining 35 enrolled Veterans were considered non-completers because they either attended less than 10 classes (24 Veterans) or did not have final assessment data for either the DJGS or 30CST (31 Veterans). See Figure 1 for a summary of the recruitment process. No enrollee experienced an injury or other significant adverse event during the classes and no enrollee reported experiencing any adverse event between classes that could have been related to their participation. Some enrollees participated from the seated position or occasionally did not complete some movements if they and their instructor felt that it would make their participation easier and safer. These decisions were made on a case-by-case basis based on the enrollee’s preference and the instructor’s judgment. 

Enrolled OVs who completed the program varied in age from 61 to 94 years old. Most completers were male (85.1%), Caucasian (67.6%), non-Hispanic (83.8%), and living independently (89.2%) with a partner (40.5%). At baseline, most completers self-rated their health as fair (27.0%) or good/very good (29.7%). Demographics did not differ significantly between the 74 enrollees who completed the program and the 35 non-completers (Table 1).

### 3.2. Change in De Jong Gierveld Score (DJGS)

**Figure 2:**
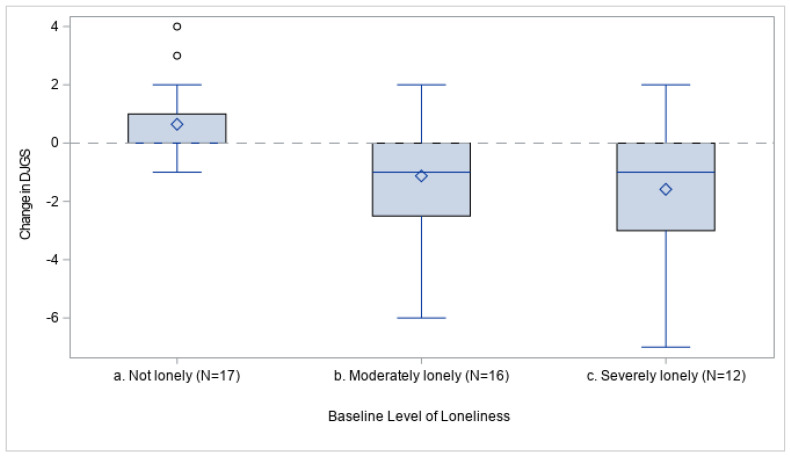
Boxplots showing distribution of change in DJGS by baseline level of loneliness.

Forty-five OVs completed a baseline and final DJGS test. At baseline, 17 (37.8%) of these OVs were not lonely, 16 (35.6%) were moderately lonely, and 12 (26.7%) were severely lonely according to the criteria developed by van Tilburg et al. [52] When the change in DJGS was examined using stepwise GLM with the transformed baseline DJGS score, site, and percent of classes attended as predictors, only the baseline score entered the model. OVs who were classified as moderately or severely lonely at baseline were significantly more likely to see a decrease in DJGS compared to those who were not lonely (model R^2^ = 0.203, *p* = 0.005) (Table 2). Based on the parameter estimates, OVs who were moderately lonely at baseline saw an average decrease of 1.13 points in their DJGS scores from baseline to the final test; those who were severely lonely saw an average decrease of 1.58 points; and those who were not lonely saw an average increase of 0.65 points (Table 2 and Figure 2). There were two outliers for change in DJGS among those with a baseline loneliness of “not lonely” (see Figure 2). After removing these two outliers and rerunning GLM, the final model was essentially the same. 

The top and bottom of each box represent the third and first quartiles of change in DJGS, respectively, for each baseline level of loneliness, with outliers shown as circles. Diamonds represent the mean change in DJGS for each baseline level. A gray dashed line representing zero change is provided for reference. Changes in DJGS below zero represent an improvement in loneliness scores from baseline to final testing, while those above zero represent a worsening of loneliness. Baseline level of loneliness was determined according to the criteria developed by van Tilburg et al. [52].

Because there is no established minimum clinically important difference for DJGS, we also examined the change in loneliness measured by ordinal categories. As shown in Table 3, most OVs (32; 71.1%) did not change their level of loneliness. Among those who did change their level of loneliness, the number that moved to a lower level of loneliness (i.e., improved) did not differ statistically from the number that moved to a higher level based on Krauth’s exact test of symmetry (nine vs. four, Chi-square = 2.50, df = 3, *p* = 0.597). 

### 3.3. Change in 30-Second Chair Stand (30CST)

Sixty-nine OVs completed the 30CST baseline and final assessments. The mean baseline score was 9.99 ± 4.60 stands/30 s and the mean final score was 12.30 ± 5.58 stands/30 s. Based on stepwise GLM, site, baseline 30CST value, age, site, and number of classes attended were not significant predictors of change in the 30CST. Therefore, the final model included only the intercept of 2.31 stands/30 s (SE = 0.376, *p* < 0.001), which is equivalent to the mean increase in 30CST among the completers, independent of site, baseline value, and attendance. 

### 3.4. Course Evaluation Survey

After finishing the virtual Tai Chi program, 43 OVs from the Puget Sound or Little Rock sites completed the course evaluation survey. None of the OVs enrolled at Palo Alto completed this survey due to an administrative error. 

In the survey, OVs were asked to rate their agreement with each of five statements on a scale from 1 (lowest agreement) to 5 (highest). Most Veterans indicated strong agreement with all five statements in the course evaluation, producing a mean course rating of 4.58 (±0.64) (Table 4). Only five Veterans expressed disagreement (i.e., a score of 1 or 2) with at least one statement. Twenty-two Veterans also provided open-ended comments, which were manually coded for themes. These open-ended comments showed that OVs were pleased with the class, the instructor, and other staff, and they expressed gratitude. Many respondents reported a benefit from the Tai Chi program, such as better balance, greater confidence, or more opportunities for social engagement. Some respondents felt that the Tai Chi program was as beneficial or more beneficial than traditional therapies. 

Very few Veterans gave negative feedback. Most negative experiences reported by respondents related to the logistics of the virtual format, such as the inability to mute other participants. One respondent felt that the instructor was unable to detect and correct participants’ mistakes on camera and suggested an in-person assessment at the beginning of the course to learn the basic movements.

### 3.5. Select Quotes from Veterans

“My balance is better than it has been in 5 years. The instructors were kind and encouraging at all times”.

“I enjoyed participating in this group. It presented an opportunity to engage in an activity with other like-minded individuals who are working towards the same or similar goals”.

“Tai Chi as well as other healing methods such as massage, naturopathic, chiropractic, acupuncture, any other methods should be offered to us vets. They are just as effective as Western medicine”.

## 4. Discussion

The purpose of this QI initiative was to provide an intervention to support OVs who were at risk of deconditioning and becoming lonely during and subsequent to the COVID-19 pandemic. We also wanted to demonstrate the feasibility and effectiveness of providing Tai Chi classes virtually. Our specific goals were to demonstrate the feasibility of establishing the program at three different VA healthcare facilities (stations) and to determine if participation in the program would yield physical benefits (i.e., improvement in 30CST), decrease feelings of loneliness, and provide an enjoyable experience for OVs comparable to what had been reported in prior research studies and other virtual Tai Chi programs [12,28,29,30,31,32,33,37]. In this regard, this initiative was successful; we demonstrated that the Tai Chi program conducted by video telehealth (using VVC) out of three different VA healthcare facilities (stations) was both feasible (based on predefined criteria) and effective.

This initiative focused on loneliness and physical deconditioning for the reasons summarized in the Introduction; both conditions had become highly prevalent among OVs as a consequence of the COVID-19 pandemic and its aftermath and were known to be associated with an increased risk of adverse clinical outcomes among older adults [6,7,8,9,10,11,12,13,14,15,19,20,21,22,23,24,25,26,27,47]. The efficacy of Tai Chi to reduce loneliness and improve physical performance was known [12,18,28,29,30,31,32,33,34]. The objective of this initiative was to use Quality Improvement methodologies to establish an effective Tai Chi program that was available to OVs using VVC. To this end, the initiative was successful. 

As a measure of effectiveness, we examined changes in DJGS and 30CST. However, we limited the analyses to OVs who had attended at least 10 classes (equivalent to 5 weeks) based on previous research that suggested that clinically meaningful changes could be detected after the 5th week [51]. On average, this subgroup of attendees (the ‘completers’) demonstrated improvement in the 30CST, which is a valid measure of physical performance and fall risk [47,48,49,50]. At final testing, these OVs were able to complete a mean of 2.31 additional stands/30 s compared to baseline. These results are consistent with the results from a non-randomized study by Li et al., which found a mean improvement of 2.07 stands among 15 older adults with mild cognitive impairment after participating in a 24-week virtual Tai Chi program [33]. Importantly, this improvement in the intervention group was statistically greater than the change noted in the study’s control group, which was assigned stretching exercises (between-group difference in mean changes (95% CI) in stands 1.87 (1.15 to 2.58)). Our results are also consistent with the weighted mean difference of 2.36 stands (95% CI 1.50–3.21, *p* < 0.00001) between the treatment and control arms reported in a meta-analysis of 11 randomized control trials of Tai Chi interventions involving frail or sarcopenic older adults [19]. None of these trials were conducted virtually and they varied from 8 to 48 weeks in duration. In our program, the site was not a significant factor when predicting changes in the 30CST (or DJGS), suggesting that Tai Chi may be equally effective across subpopulations and independent of the instructors and other staff implementing the intervention. 

Studies of various patient populations have reported minimal clinically important (MCID) differences for the 30CST that range from 2.0 to 2.6 stands/30 s; three of the four methods utilized in these studies for this calculation determined the MCID to be 2.0 stands/30 s [53,54]. The participants completing our program improved their 30CST by an average of 2.31 additional stands/30 s. Based on the most commonly reported MCID, this is a clinically significant change. This finding provides further evidence that a Tai Chi course conducted virtually is an effective, clinically significant intervention for older patients.

OV attendees who were experiencing loneliness at the time of entering the program also benefited from participating in the Tai Chi classes. Those who were moderately or severely lonely at baseline saw a mean improvement (decrease) of 1.13 and 1.58 points in the DJGS, respectively. Although there is no established MCID for DJGS, these results are similar to those reported by Chan et al. based on an RCT comparing Tai Chi with usual care in a group of socially isolated older adults [18]. The subjects randomized to Tai Chi twice a week for three months experienced a 1.9-point decrease in their scores on the shortened, six-item version of the DJGS, which was significantly greater than the 0.5-point decrease experienced by the usual care control group. The similarity of our results to those reported by Chan et al. provides strong evidence that group Tai Chi classes have positive benefits for older adults who are lonely. Whether this effect is due to some aspect of Tai Chi, such as the focus on meditation, or the result of participating in group classes is unknown. 

The effects of participation in different types of group exercises on older subjects’ social well-being, including loneliness, has been examined in prior studies [55,56]. In these studies, the investigators found that the older subjects’ feelings of loneliness decreased after 6 to 12 months of training. They also found that the level of social support that the subjects felt was provided by their groups was directly associated with their improvement in feelings of loneliness, independent of the type of exercise their assigned group performed, whether stretching, dancing, or walking. These results suggest that the benefits of exercise on psychological outcomes may be partially related to social interactions inherent in group-based programs. However, all the exercise classes were conducted in person and it was not stated whether any effort was made to promote social interaction and comradery within any group during the exercise classes. Further research is needed comparing in-person and virtual exercise programs and Tai Chi to other types of group exercises on loneliness among older adults. 

Because the current literature shows no established guidelines for what is considered a clinically meaningful change in DJGS, the frequency with which the OVs moved from one loneliness level (as defined by van Tilburg et al. [52]) to another between baseline and final testing was also examined. Most OVs did not see a large enough change in DJGS to place them in a different level of loneliness at final testing. Those who did shift to a different level were just as likely to move to a worse compared to a better level of loneliness. Consequently, more work is needed to determine how best to assess the clinical significance of any statistically significant change in the DJGS. 

As a measure of program feasibility, we utilized the criteria established by Sawyer et al. [37]. Consistent with these criteria, we found that the majority of the participants were able to (1) connect and actively participate in the classes using VVC; (2) subsequently reestablish connections adequate to participate in at least 10 classes with little or no technical difficulties; and (3) participate from home without developing safety concerns. In keeping with the final feasibility criteria, many OVs who finished the program provided glowing assessments of the program and none indicated having any significant objections or concerns. Overall, the program ran very smoothly. As planned, the program was successfully implemented at all three participating sites providing OVs the opportunity to safely attend the Tai Chi classes from home using VVC. Although the amount of startup support provided to the participants was not recorded in a consistent manner, staff notes indicate that many Veterans with limited or no previous experience using VVC were able to learn how to navigate the VVC platform and participate in group virtual classes, aided by equipment loans and technical assistance when necessary. The VVC-based virtual Tai Chi program also seemed to appeal to a wide range of older Veterans, as program completion in this sample did not differ significantly by any demographic measures. Most of the enrolled OVs maintained excellent attendance and completed the program without any major safety concerns or adverse events being reported. Course evaluation results indicated that the participants perceived the program as therapeutic and enjoyable, leaving them motivated to continue practicing Tai Chi.

There have been four other recently published summaries of virtual Tai Chi programs that complement the findings from our initiative [33,36,38,39]. None of these programs were conducted within the VA or specifically targeted OVs. As mentioned above, the study by Li et al. was a randomized efficacy trial comparing Tai Chi with stretching exercises [33]. Of the 30 subjects enrolled, 15 were assigned to Tai Chi. All subjects had mild cognitive impairment, were 65 years of age or older, participated virtually by Zoom, and were non-Veterans. In addition to reporting similar findings on the 30CST for the Tai Chi group, their compliance rate was roughly the same as ours; less than half the participants attended 80% or more of the classes. The study by You et al. was a non-randomized efficacy trial comparing Tai Chi (n = 21) with light exercise (n = 11) [39]. Their completion rate was similar to what we found in our initiative, although it was unclear how this term was defined. No other outcomes were reported. Most of the subjects in the Tai Chi group were older black (53%) females (66%) from an urban setting. Lee et al. reported the results of a well-designed QI initiative to establish a virtual program of Tai Chi for Arthritis and Fall Prevention for Older Adults During COVID-19 [36]. Of the 83 participants, 61 (73.5%) were reported to have completed the 8 week/16 class program. However, this term was not defined, and no attendance results were reported. All participants were older than 65 years, and most were female (>80%), white (>92%), and college graduates (>85%). No clinical outcomes were reported. The other virtual Tai Chi program was that reported by Staller et al. [38]. This program was unlike ours in that it focused on young patients with irritable bowel syndrome. Of the 109 OVs who formally enrolled in our virtual Tai Chi program, 74 (68%) completed the program after attending a mean 20.1 (83.8%) of 24 possible classes (Table 1). In total, all of these reported results highlight the challenges of maintaining high attendance rates among older participants in these types of virtual exercise programs.

We also found that promoting comradery and socialization presented more of a challenge than we had expected. You et al. addressed this issue in their virtual Tai Chi program by scheduling two breaks for socialization and discussion within each class [39]. Our original intention was to provide similar socialization breaks; however, audio and video issues limited this plan. It was necessary to mute the participants’ microphones for much of the sessions as it was difficult to understand what anyone was saying (including the instructor) when more than one person spoke simultaneously. Communication had to occur in an organized fashion with project staff managing microphones. Project staff also had to manage the view shown on screen and spotlight the instructor or participant when needed. The person spotlighted would take up most of each participant’s screen, while the remaining participants would be seen in smaller viewing panes at the bottom of the screen. At the time of this program, VVC did not allow participants to have independent control of the images shown on their device. Most often, participants would only see the instructor spotlighted, while the instructor saw only one participant spotlighted with all other Veterans still visible on screen. Had it been possible to address the audio and visual issues more effectively, the impact of participation on loneliness may have been even greater. Despite these challenges, the OVs found the course to be very rewarding.

### Limitations

This paper has several important limitations. One such limitation is the lack of information about the OVs referred to the program. We did not keep records of the number of referrals or the barriers that caused some OVs to decide against enrolling or to withdraw after starting the program. We were successful in formally enrolling over 100 OVs into the program, and program completers did not report experiencing any major technical problems keeping them from participating in the virtual sessions. However, the participants may not have represented the population of Ovs living within the catchment areas of the three participating VA healthcare facilities. Of particular importance in this regard, it is known that both a low income and living in a rural setting are associated with less access to broadband services [57,58]. For this initiative, we were not able to collect these parameters from the OVs referred to our program. In the future, it will be important to include an examination of rurality and other factors known to be related to virtual care access when assessing the feasibility of conducting virtual exercise program for OVs. It is these disadvantaged OVs who theoretically could benefit the most from such a program, but only if they have adequate access. 

The recruitment strategy used In this initiative was a second potential limitation. The program was open to all OVs who felt, or who their healthcare providers felt, could benefit from participation. Not all of the sites selectively recruited older Veterans with signs of diminished lower extremity physical function or feelings of loneliness. As several of the new enrollees scored well on the baseline 30CST and/or the DJGS, there was limited potential for these individuals to demonstrate improvement in these instruments upon completion of the Tai Chi course. We attempted to mitigate this problem by including baseline measures in the GLM analyses. Interestingly, the 30CST improved for most OVs independent of their baseline value, suggesting that our program is beneficial even to those without diminished lower extremity physical function. Our analysis of DJGS was also limited by the relatively large number of missing responses; only 45 out of 75 enrolled OVs completed baseline and final DJGS assessments. As expected, we did not demonstrate a statistically significant deterioration in either outcome measure among the course completers.

Tests for differences in program completion by race, marital status, household composition, and self-rated health at baseline may have been underpowered due to small counts within subcategories. Interpretation of these tests is also limited by our definition of completion. For example, some of those who did not “complete” the program attended as many as 21 classes yet were classified as non-completers because they lacked baseline or final assessment data. Although this is an issue that we have identified in other clinical and research initiatives, we are sometimes unable to determine why some OVs attend all exercise sessions but elect not to complete the final post-course assessments. 

There were potentially many other factors that were not measured in this project that could have impacted the quality and effectiveness of the virtual classes. For example, we did not compare the types of equipment that the OVs used, or attempted to use, for linking to the classes through VVC. The equipment to which they had access (e.g., smartphones, tablets, small desktop computers) may not have been ideally suited for this purpose. As suggested by Gell et al. [59], we provided ongoing technical support to the participants and encouraged family members to assist as well. As described in the Section 2, we also helped OVs wishing to join the program to obtain better equipment whenever possible. Despite the assistance we provided, a couple of OVs continued to have problems participating virtually. None of these OVs completed the program. It is not known how many OVs desired to join our virtual Tai Chi program but did not do so because of such problems. In a study in which 32 subjects were assigned to either Tai Chi or light exercise groups conducted virtually, You et al. determined that many of the participants had relatively low scores on the Attitudes Towards Computers Questionnaire [39,60], which indicated they were not very comfortable using computers. They also reported that most participants who withdrew from the study did so primarily due to perceived technological barriers.

Although not a true limitation, it must be kept in mind that this was a clinical Quality Improvement initiative, not a research study. For this reason, the project was not driven by an a priori hypothesis, there was no randomization to different interventions, and there was no comparison group. The data analyses were conducted so as to provide evidence as to whether the participants experienced benefits that were both statistically significant and clinically meaningful. The results presented are consistent with the findings from prior randomized controlled clinical trials and help to justify the continued financial support of the clinical initiative.

## 5. Conclusions

This project indicates that a virtual Tai Chi program is a valid alternative to in-person classes for OVs at risk of loneliness or physical deconditioning. OVs were able to navigate VVC, showed good rates of attendance and program completion, demonstrated improvements in physical performance and loneliness, and reported positive experiences. All three sites showed similar effectiveness and feasibility, with no detectable differences in completion rates based on participant demographics. Overall, our results suggest that a virtual intervention such as this Tai Chi program can be successfully implemented through VVC, which may play an important role in expanding access to services for OVs who are unable to attend therapeutic programs in person.

## Figures and Tables

**Table 1:** Completion status by attendance and demographics of enrollees.

Demographic	Program Completers	Non-Completers	*p*-Value *
Total enrollees, n	74	35	
Number of classes attended, mean (±SD) [min, max]	20.1 (±4.12) [10, 24]	7.3 (±5.64) [1, 21]	
Site, n (%)			
Little Rock	20 (62.5)	12 (37.5)	0.512
Palo Alto	27 (75.0)	9 (25.0)	
Puget Sound	27 (65.9)	14 (34.1)	
Age, mean (±SD)	72.0 (±6.80)	71.0 (±6.24)	0.483
Gender, n (%)			
Male	63 (72.4)	24 (27.6)	0.128
Female	11 (55.0)	9 (45.0)	
Unknown gender	0 (0.0)	2 (100.0)	
Race, n (%)			
Caucasian	50 (65.8)	26 (34.2)	0.793
Black or African American	7 (63.6)	4 (36.4)	
Another race	4 (80.0)	1 (20.0)	
Unknown race	13 (76.5)	4 (23.5)	
Ethnicity, n (%)			
Not Hispanic or Latino	62 (69.7)	27 (30.3)	0.086
Hispanic or Latino	2 (33.3)	4 (66.7)	
Unknown ethnicity	10 (71.4)	4 (28.6)	
Marital status, n (%)			
Married or domestic partner	41 (73.2)	15 (26.8)	0.497
Divorced, separated, or widowed	20 (62.5)	12 (37.5)	
Single, never married	6 (60.0)	4 (40.0)	
Unknown marital status	7 (63.6)	4 (36.4)	
Residential status, n (%)			
Living independently	66 (68.0)	31 (32.0)	1.000
Living with private person	4 (80.0)	1 (20.0)	
Unknown residential status	4 (57.1)	3 (42.9)	
Household composition, n (%)			
Lives with only spouse/partner	30 (65.2)	16 (34.8)	0.486
Lives alone	19 (70.4)	8 (29.6)	
Lives with spouse/partner and children	8 (88.9)	1 (11.1)	
Other household composition	10 (58.8)	7 (41.2)	
Unknown household composition	7 (70.0)	3 (30.0)	
Self-rated health at baseline, n (%)			
Poor	3 (42.9)	4 (57.1)	0.273
Not so good	10 (62.5)	6 (37.5)	
Fair	20 (71.4)	8 (28.6)	
Good	19 (82.6)	4 (17.4)	
Very good	3 (60.0)	2 (40.0)	
Unknown self-rated health	19 (63.3)	11 (36.7)	

* *p*-value based on *t*-tests for continuous data and Chi-square or Fisher’s exact tests for nominal data, after excluding those with unknown values. Note: tests may be underpowered for race, marital status, household composition, and self-rated health at baseline.

**Table 2:** Change in DJGS by baseline category of loneliness *.

Parameter	β Estimate	Standard Error	*p*-Value
Intercept	0.647	0.483	0.188
Severely lonely at baseline (DJGS 9 to 11) **	−2.230	0.751	0.005
Moderately lonely at baseline (DJGS 3 to 8) **	−1.772	0.694	0.014
Not lonely at baseline (DJGS 0 to 2)	0		

* Baseline level of loneliness according to the criteria developed by van Tilburg et al. [52]. **: significant at α = 0.05.

**Table 3:** Final level of loneliness by baseline level of loneliness *.

	Final Level of Loneliness			
Baseline Level of Loneliness	Not Lonely (DJGS 0 to 2)	Moderately Lonely (DJGS 3 to 8)	Severely Lonely (DJGS 9 to 11)	Total
Not lonely (DJGS 0 to 2)	14	3	0	17
Moderately lonely (DJGS 3 to 8)	5	10	1	16
Severely lonely (DJGS 9 to 11)	1	3	8	12
Total	20	16	9	45

* Note: Cell values indicate number of Veterans with given baseline and final loneliness level. Green cells represent improvement in DJGS loneliness level, orange cells represent worsening, and unshaded cells represent no change.

**Table 4:** Course evaluation survey.

Statement	N	Mean	Std Dev	Min	Median	Max
I found the Tai Chi course to be very rewarding.	43	4.58	0.85	2.00	5.00	5.00
The Tai Chi instructor was knowledgeable, prepared, and effective in helping me reach my personal best.	43	4.81	0.63	2.00	5.00	5.00
I plan to continue to practice Tai Chi on my own or with a group.	43	4.42	0.88	2.00	5.00	5.00
I feel that the Tai Chi course was of benefit to my health, and it improved my walking and balance.	42	4.50	0.97	1.00	5.00	5.00
I was treated with kindness and respect during my Tai Chi classes.	43	4.84	0.69	1.00	5.00	5.00
Total score (average of all questions)	43	4.61	0.64	1.80	4.80	5.00

## Data Availability

Due to US Department of Veterans Affairs (VA) regulations and our ethics agreements, the analytic datasets used for this study are not permitted to leave the VA firewall without a Data Use Agreement. This limitation is consistent with other studies based on VA data.
